# Cooperativity of *Rb*, *Brca1*, and *p53* in Malignant Breast Cancer Evolution

**DOI:** 10.1371/journal.pgen.1003027

**Published:** 2012-11-15

**Authors:** Prashant Kumar, Malini Mukherjee, Jacob P. S. Johnson, Milan Patel, Bing Huey, Donna G. Albertson, Karl Simin

**Affiliations:** 1 Department of Cancer Biology, University of Massachusetts Medical School, Worcester, Massachusetts, United States of America; 2 Department of Pediatric Hematology/Oncology, Texas Children's Cancer Center, Baylor College of Medicine, Houston, Texas, United States of America; 3 Helen Diller Family Comprehensive Cancer Center, University of California San Francisco, San Francisco, California, United States of America; Baylor College of Medicine, United States of America

## Abstract

Breast cancers that are “triple-negative” for the clinical markers ESR1, PGR, and HER2 typically belong to the Basal-like molecular subtype. Defective *Rb*, *p53*, and *Brca1* pathways are each associated with triple-negative and Basal-like subtypes. Our mouse genetic studies demonstrate that the combined inactivation of Rb and p53 pathways is sufficient to suppress the physiological cell death of mammary involution. Furthermore, concomitant inactivation of all three pathways in mammary epithelium has an additive effect on tumor latency and predisposes highly penetrant, metastatic adenocarcinomas. The tumors are poorly differentiated and have histologic features that are common among human *Brca1*-mutated tumors, including heterogeneous morphology, metaplasia, and necrosis. Gene expression analyses demonstrate that the tumors share attributes of both Basal-like and Claudin-low signatures, two molecular subtypes encompassed by the broader, triple-negative class defined by clinical markers.

## Author Summary

These studies establish a unique animal model of aggressive forms of breast cancer for which there are no effective, targeted treatments. *Rb*, *p53*, and *Brca1* are associated with inherited forms of cancer, but defects in these pathways are also found together in a subset of breast cancer patients without a family history of the disease. Simultaneous inactivation of all three pathways causes more aggressive disease than do pair-wise combinations, indicating that the pathways play non-overlapping roles in tumor prevention.

## Introduction

The dire need for more effective treatments for aggressive breast cancers has motivated intensive investigations into their cellular and molecular etiology. Breast cancers classified as “triple-negative” by clinical diagnostic markers (ESR1, PGR, and HER2 negative) are heterogeneous in their clinical behavior, morphology, and molecular biology. Triple-negative breast cancers (TNBC) typically express the Basal-like molecular signature, thus TNBC and Basal cancer classifications are frequently used interchangeably. However, they are not completely synonymous [1], [2]. TNBCs also include the Claudin-low molecular subtype [3], which is characterized by greatly reduced expression of intercellular junction components and by activation of molecular pathways associated with epithelial-to-mesenchymal transition (EMT), cancer stem cells, and the immune response [4].

Histologically, most triple-negative breast cancers are invasive ductal carcinomas, but TNBCs also include the metaplastic, medullary, and adenocystic histologic special types, distinctive morphologies that are prevalent among Claudin-low tumors [4]. TNBCs are insensitive to endocrine therapy and HER2 antagonists, but they *are* sensitive to chemotherapy. Nevertheless, long-term patient outcomes are poor due to high rates of relapse and acquired chemoresistance [5], [6]. Mouse models that mimic the complexity of TNBC will be invaluable tools for defining the diverse cellular biology and behavior of these tumors and for rigorously triaging new drug candidates.

Basal-like breast cancers often simultaneously inactivate three tumor suppressors that are infamous for their roles in familial cancers: *Rb* (*RB1*) [7], *p53* (*TP53*) [8], and *BRCA1* [9]. *p53* is mutated in 20–30% of human breast cancers and defective pathway intermediates also increase breast cancer risk [10]. Moreover, nearly all Basal-like cancers with *BRCA1* mutation have concomitant *p53* mutation [11]. Germline *BRCA1* mutation predisposes early-onset breast cancers that are often triple-negative and that correlate with Basal-like tumors in microarray analyses [9]. *BRCA1* mutation accounts for nearly half of familial breast cancers (OMIM 604370), but *BRCA1* is also down-regulated in sporadic breast tumors without germline mutation [12]. The overall effect of BRCA1 loss is likely pleiotropic. The best studied function of BRCA1 is in orchestrating DNA double-strand break repair through homologous recombination or non-homologous end-joining. The importance of defective DNA repair in breast cancer is underscored by the observation that all known genes associated with inherited forms of the disease safeguard genomic integrity [13]. BRCA1 also functions as a transcription factor [14] that appears to be required for the differentiation of stem/progenitor cells into mature luminal cells [15]. This finding is consistent with the recent characterization of *BRCA1*-associated tumors as aberrant luminal progenitor cells [16].

The pair-wise cooperativity of *Brca1* and *p53* in mammary tumorigenesis has been studied in mouse models [17]. We and others have investigated the impact of combined *Rb* and *p53* inactivation on mammary tumorigenesis *in vivo* [18]–[20]. We showed that following *Rb* perturbation *in vivo*, tumor progression is limited largely by p53-dependent apoptosis, and that loss of the second *p53* allele in *MFT_121_*+/*p53^f/+^* tumors is likely a prerequisite for mammary tumor progression [19], since the vast majority of tumors lose the wild type *p53* allele during tumorigenesis. In the context of a brain carcinoma model initiated by T_121_, apoptosis appears to be the critical function of the normal *p53* allele that is the target of selective pressure [21].

Our motivation to combine *Rb* inactivation with *Brca1* and *p53* mutation derives, in part, from the observation that the *Rb* gene is among the most frequently deleted loci in *Brca1*/*p53*-mutated mouse tumors [22], indicating that *Rb* is a critical barrier to tumor progression. *Rb* pathway inactivation is strongly associated with human triple negative breast cancers. A cardinal feature of basal-like breast cancers is the abundant expression of the “proliferation cluster” genes [7], which include many E2F-regulated genes that are de-repressed following Rb inactivation. In human breast cancers, reduced pRb activity correlates with higher tumor grade [23], but also predicts improved chemotherapy responsiveness [24]. The *Rb* gene itself is mutated in breast cancer [25], and recent genomic studies have indicated an overrepresentation of mutations within pRb-binding sites of human gene regulatory domains [26].

In this study we show that mammary tumors caused by inactivation of the *pRb* family (pRb_f_) of proteins (pRb, p107, p130), together with *Brca1* and *p53* inactivation, mimic several aspects of the most aggressive forms of breast cancer, including rapid tumor progression, poor differentiation, distant metastasis, necrosis, metaplasia, and genomic instability. Our findings illustrate the compounding effect of acquiring multiple tumor suppressor mutations during tumor evolution and underscore the distinct requirements of each of these canonical tumor suppressor proteins.

## Results

### Conditional T_121_ expression in mammary epithelium

**Figure 1:**
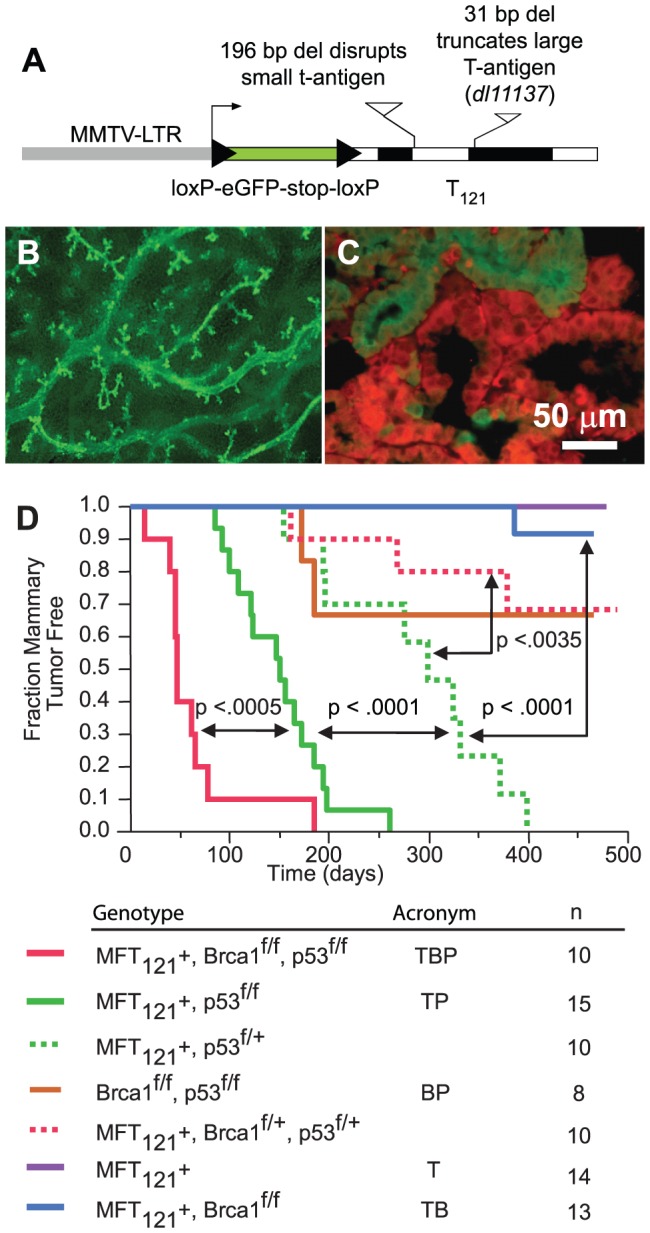
The *MFT_121_* transgene construct. (A) The *MFT_121_* transgene construct. The “floxed” eGFP-stop cassette was expressed throughout virgin mammary epithelium (B, original magnification 50×). Following Cre-induced excision, T_121_ was expressed (red) in the majority of luminal epithelial cells (C). eGFP immunolabeling (green) revealed non-recombined cells (original mag. 400×). Kaplan-Meier analysis of tumor onset (D). *p53* was haploinsufficient (dashed green) for tumor suppression (p<0.0001, log-rank test). Homozygous *p53* mutation (solid green) shortened tumor latency. *Brca1* loss (solid red) further accelerated tumor onset (p<0.0005). Median tumor latency of TBP mice was approximately seven weeks. Parturition Day 1 = Time 0. All mice harbored the Wap-Cre transgene (not shown). Significance levels for critical comparisons are indicated.

We constructed the *MFT_121_* (MMTV-Floxed-eGFP-T_121_) transgene to conditionally inactivate the pRb family (pRb_f_) of pocket proteins in mammary epithelium (Figure 1A). The T_121_ protein is an amino-terminal fragment of the SV40 large T antigen that perturbs pRb_f_ activity and predisposes tumorigenesis in a range of tissues [27]. While *Rb* inactivation alone is sufficient to induce mammary tumors [20], the shorter latency of *TgWAP-T_121_* tumors indicates there is functional redundancy or compensation by the related pocket proteins p107 or p130 [19]. In the *MFT_121_* model, the MMTV-LTR promotes mammary-specific transgene expression (Figure 1B). The approach of inactivating pRB_f_, Brca1, and p53 specifically in mammary epithelial cells via the *Wap-Cre* transgene [28] enabled us to avoid the appearance of lymphomas [20] or sarcomas [29]. Wap-Cre excised the LoxP-eGFP-stop-LoxP reporter cassette and initiated *T_121_* expression in ductal and alveolar luminal epithelial cells (Figure 1C). Virgin glands of MFT_121_; WAP-Cre mice appeared normal. Lactating glands (WAP-Cre-induced) showed reduced alveolar density, similar to the gland atrophy phenotype in the *TgWap-T_121_* model, which was associated with apoptosis caused by T_121_-induced proliferation [19] and lactation defects observed in WAP-Cre; *Rb^fl/fl^*; *p107^−/−^* mice [20].

### Concomitant *pRb_f_*, *p53*, and *Brca1* inactivation significantly accelerates tumor onset

Multiparous *TgMFT_121_*; *TgWap-Cre* mice remained tumor-free for more than a year after Cre-induction, but mice that were either heterozygous or homozygous for a conditional *p53* allele [30] (*TgMFT_121_; TgWAP-Cre; p53^f/+^* or *TgMFT_121_; TgWAP-Cre; p53^f/f^*) developed mammary tumors with 100% penetrance and clear evidence of *p53* haploinsufficiency (Figure 1D, p<0.0001, log-rank test). Heterozygous *p53* mice developed tumors with a median latency of 299 days, while *p53* homozygous mice had a median tumor latency of 150 days. These results are consistent with previous findings that *p53* activity is rate limiting for mammary tumor progression initiated by *Rb* inactivation [18]–[20]. In contrast to the high penetrance we previously found in our *TgWAP-T_121_* breast tumor model [19], the present studies indicate that the *TgMFT_121_* transgene only partially inhibits pRb_f_ pathways, possibly owing to the reduction in the transgene gene copy number following Cre excision. Similar dosage effects were observed in a conditional *T_121_* transgenic model of astrocytoma [31].

*Brca1* mutation dramatically accelerated tumor onset in mice with Rb_f_/p53 inactivation (Figure 1D, p<0.0005). Only a single mouse (n = 13), doubly defective for *Rb_f_* and *Brca1* activity (*TgMFT_121_;TgWAP-Cre;Brca1^f/f^*), developed mammary tumors at 386 days following Cre induction by multiple pregnancies. In contrast, 100% (n = 10) of *TgMFT_121_*; *Brca1^f/f^ p53^f/f^*; *TgWAP-Cre* mice (hereafter, TBP) developed mammary tumors with a median latency of only 47 days. Thus, a single pregnancy was sufficient for 100% TBP tumor penetrance. Once palpable, TBP tumors grew rapidly to 1500 cc within several days (data not shown). Therefore, inactivation of the three canonical tumor suppressors showed additive effects on tumor latency, since TBP mammary tumors developed significantly faster than did pair-wise combinations.

### Enhanced survival and increased proliferation rates cause rapid tumor progression in *pRb_f_*/*p53*/*Brca1*-perturbed epithelium

**Figure 2:**
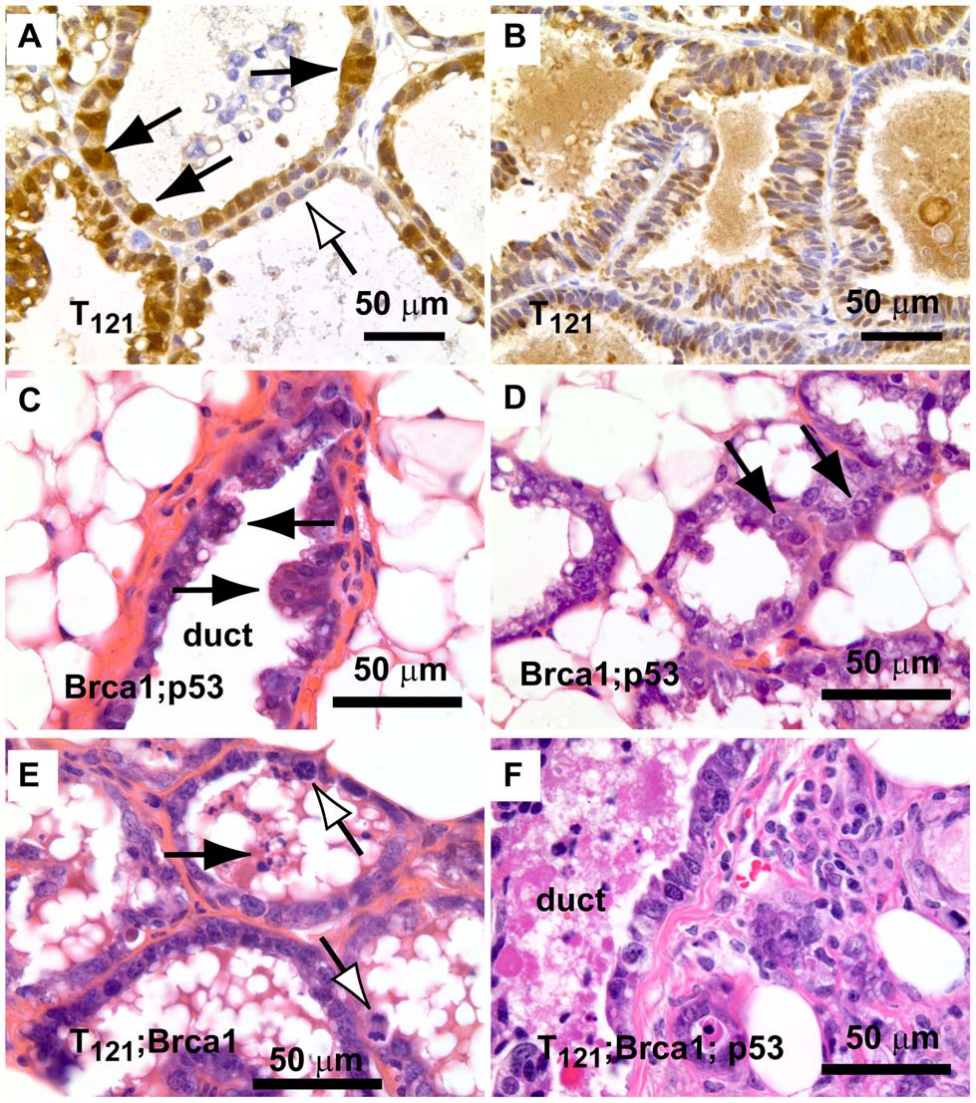
Early lesions in lactating mammary glands. (A) Nuclear and cytoplasmic T_121_ expression (brown) is associated with enlarged, pleiomorphic nuclei (filled arrows) compared to low-expressing cells (open arrow). (B) Benign, multi-layered, mammary intra-epithelial neoplasia (MIN) within the same gland as A. (C) Dual inactivation of *Brca1* and *p53* disrupted epithelial architecture in primary ducts (C) and lactating alveoli (D), shown by papillary tufting (C, arrows) and high grade nuclei and prominent nucleoli (D, arrows). High grade nuclei and mitotic figures (E, open arrows) and dead cell debris (arrow) in *MFT_121;_ Brca1* gland. Severely pleomorphic and high grade nuclei are visible throughout *MFT_121;_ Brca1; p53* gland (F).

**Figure 3:**
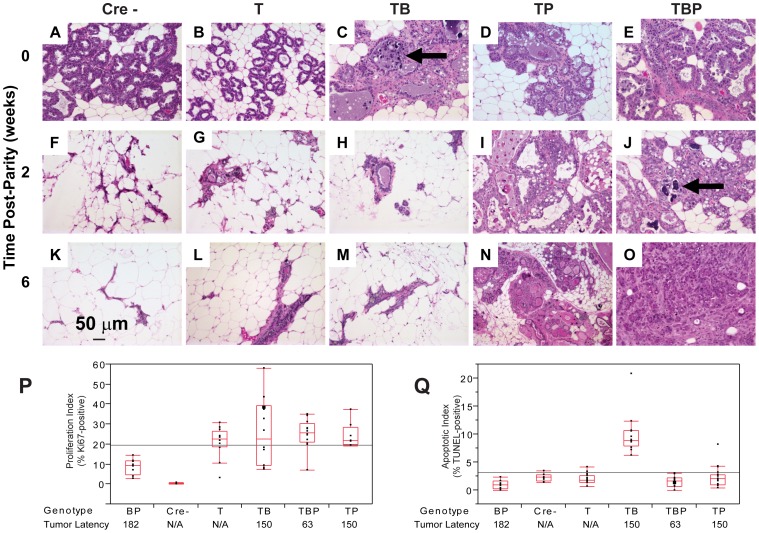
Combined inactivation of pRb_f_ and p53 causes a durable block in involution. Lactating mammary epithelium of Cre-negative control mice (A, F, K) and *T_121_*-expressing mice (B, G, L) involute normally (A–E: 0 wks, F–J: 2 wks, K–O: 6 wks). T_121_-expressing mice have reduced alveolar density. Cell death and debris were abundant in TB glands (arrows panels C, J). TP glands failed to involute (N) and persisted as highly cystic glands. Frank tumors were present by 6 wks in TBP mice. An invasive adenocarcinoma fills the field of panel O. Measured at 0 wks, *T_121_* increased the Ki67 index but without added effect by *Brca1* and/or *p53* loss (P). Combined inactivation of *pRb_f_* and *Brca1* increased TUNEL-positive cells, which was suppressed by *p53* loss (Q, p<0.0001). For each genotype n = 5 mice. Original magnification of each panel was 200×. T = *T_121_*, B = *Brca1*, P = *p53*.

To investigate the early effects of tumor suppressor inactivation, we examined mammary gland biopsies (n = 5 mice) from time points (0, 2, 6 weeks) following forced weaning at Day 1 parturition. At the earliest time point, T_121_ expression alone was sufficient to cause benign, hyperproliferative lesions (Figure 2A, 2B). T_121_-expressing cells showed a higher Ki67 index than did cells without T_121_, as expected (p<0.0063, Figure 3P). Combinations with *Brca1* and *p53* mutation caused higher grade, premalignant lesions (Figure 2C–2F). Tall columnar epithelia of darkly staining cells and papillary tufting (Figure 2C) were characteristic of the disrupted epithelial morphology. Pleomorphic, faintly staining nuclei with prominent nucleoli were common among T_121_ expressing cells (Figure 3F).

The combined inactivation of *pRb_f_* and *p53* also dramatically suppressed the physiologic cell death of mammary involution (Figure 3I, 3N), although pRb_f_ perturbation alone had little effect (Figure 3B, 3G, 3L). It was shown that *p53* mutation alone delayed involution by several days [32]. Here, we observed that the combined inactivation of pRb_f_ and p53 blocked involution through six weeks (Figure 3I, 3N). Biopsies from mice harboring *Brca1* mutation (Figure 3C, 3J) showed extensive cellular debris from dead or dying cells within their lumen. The dual loss of *pRb_f_* and *Brca1* activities (*TgMFT_121_;TgWAP-Cre;Brca1^f/f^*) triggered elevated cell death that was p53-dependent (p<0.0001, Figure 3Q). The combined loss of pRb_f_, Brca1, and p53 activities accelerated tumor progression, indicated by frank tumors that appeared by six weeks (Figure 3O). However, when measured at the earliest time point, neither an increased Ki67 proliferation index (Figure 3P) nor a decreased cell death index (Figure 3Q) presaged faster onset of TBP tumors compared to *Rb_f_*/*p53* tumors. Thus, full transformation occurred in only a minority cell population, which likely reflects the requirement for additional collaborating oncogenic events.

### Histopathologic features of tumors with conditional inactivation of *pRb_f_*, *p53*, and *Brca1*

**Figure 4:**
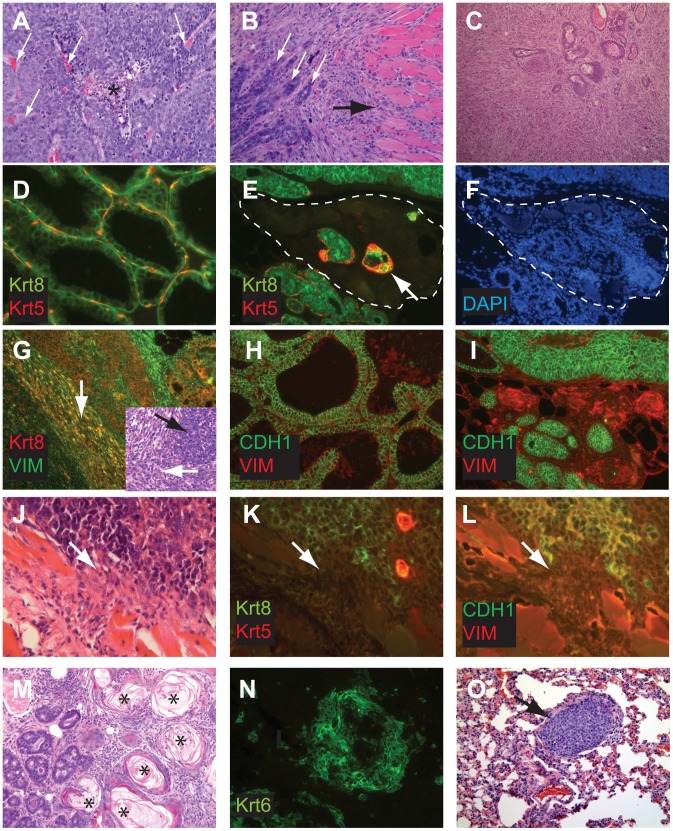
Perturbed differentiation in TBP tumors. Poorly differentiated tumors with solid morphology were most common (A), with and without vascularization (white arrows) or central necrosis (asterisk). Other tumors retained remnant glandular architecture (B, white arrows). Metaplastic cells invaded adjacent muscle and stroma (B, black arrow). Homogeneous spindloid cells of a carcinosarcoma entrap carcinomatous cells (C, 100×). Keratin-8 (Krt8, green) and Keratin-5 (Krt5, red) immunolabeling of luminal and myoepithelial cells, respectively (D, E, K). Greatly reduced expression of both Krt5 and Krt8 (E dashed lines, and K arrow). DAPI staining (blue) indicates the high cellularity of the region devoid of epithelial markers in panel E (F). Metaplastic tumor cells (G) with dual staining of Krt8 (red) and the mesenchymal marker Vimentin (green), or reduced Krt staining (K). Abundant E-cadherin (CDH1, green) in normal adjacent (H) or well-differentiated tumor (I). Reduced or absent CDH1 along invasive tumor fronts (J–L). Keratinic whorls in squamous metaplastic cells (M, asterisks). Whorl-associated and disseminated Keratin 6 expression (N, green). Pulmonary metastases were observed in both T_121_/p53 and TBP mice (O, arrow).

T_121_/p53 (TP) and TBP mice developed high grade mammary adenocarcinomas with heterogeneous phenotypes indicative of highly perturbed differentiation, summarized in Table 1. Tumors of both genotypes showed mixed solid and glandular morphologies. More TBP mice than TP mice (13/14 vs. 8/16 cases, p = 0.0169, two-tailed Fisher's exact) developed solid tumors that were largely devoid of glandular architecture (Figure 4A). All solid tumors showed a mixture of pushing and infiltrative borders, and nearly half of the tumors were vascularized (Figure 4A). All carcinomas expressed varying levels of luminal epithelial markers, including Keratin-8 (Figure 4E, 4G, 4K) and E-Cadherin (Figure 4I, 4L). These markers were abundant in well-differentiated tumor regions but were greatly diminished or absent in poorly differentiated areas (Figure 4E, 4I, 4K, 4L). Nests of carcinoma cells variably expressed basal/myoepithelial lineage markers (Keratins-5, -14, Figure 4E, 4K).

**Table 1:** Characteristics of TP and TBP tumors.

Histologic Feature	*TgMFT_121_*,*Brca1^f/f^,p53^f/f^*	*TgMFT_121_, p53^f/+^*	*TgMFT_121_, p53^f/f^*
	n = 14 (%)	n = 11	n = 16
**Solid** *	13 (93)	6 (55)	8 (50)
**Glandular**	4 (29)	2 (18)	6 (38)
**Spindloid metaplasia**	9 (64)	4 (36)	5 (31)
**Carcinosarcoma**	1 (7)	3 (27)	4 (25)
**Squamous Metaplasia**	2 (14)	2 (18)	4 (25)
**Myoepithelioma** *	4 (29)	0 (0)	0 (0)
**Cribriform**	0 (0)	1 (9)	0 (0)
**Invasion**	9 (64)	4 (36)	8 (50)
**Apoptosis/Necrosis** †	12 (86)	7 (64)	8 (50)
**Fibrosis**	1 (7)	1 (9)	5 (31)
**Angiogenesis**	5 (36)	5 (45)	7 (44)

Significant at p<0.05.

* two-tailed.

† one-tailed Fisher's exact.

Many tumors showed multiple, mixed morphologies; therefore, the percentage reflects the proportion of tumors showing the phenotype within each genotype. We saw no differences due to the initial *p53* allele status (f/+ vs. f/f). We combined these classes (n = 11+16 = 27) for statistical comparison to tumors with *Brca1* mutation (n = 14).

Both TP and TBP mice (4/16 vs. 1/14 cases, p = 0.3359) developed carcinosarcomas (Figure 4C), also known as “EMT” (Epithelial to Mesenchymal Transition) tumors [33]. These tumors are comprised of faintly-staining, fusiform, spindloid cells and are characteristic of *p53* mutant mice. The histology of hematoxylin and eosin stained carcinosarcomas is relatively homogeneous and is visibly distinct from carcinomas with spindloid metaplasia that showed biphasic carcinomatous and spindloid morphologies (Figure 4B, 4G). Among both carcinosarcomas and metaplastic tumors, we observed dual expression of mesenchymal (Vimentin) and epithelial (Keratin-8) markers (Figure 4G), which are mutually exclusive lineage markers in the normal gland. Dual expression is also a feature of human Claudin-low [4] and mouse EMT tumors [33]. Poorly differentiated spindloid cells along invasive tumor fronts showed either dual expression (Figure 4G) or greatly diminished epithelial marker expression (Figure 4J–4L).

In both TP and TBP tumors, we observed squamous metaplasia that was characterized by keratin nests (Figure 4M) and high expression of *Keratin 6* (Figure 4N), a marker of progenitor cells that is expanded in *Wnt-1*-induced mammary tumors. Four of fourteen cases (29%) of TBP tumors, but zero cases of TP tumors (p = 0.0365), included whorls of spindloid cells that resembled myoepithelial cells (not shown). Finally, central necrosis, a hallmark feature of human *BRCA1*-mutated tumors, was present in twelve of fourteen TBP cases, a greater proportion than the eight of sixteen T_121_/p53 cases (p = 0.0577), indicating that the selective pressure of cell death persists even in the absence of p53 activity.

Distant metastases were observed in the lungs of both TP (4 of 14 cases) and TBP (3 of 6 cases) mice (Figure 4P), which is noteworthy because relatively few transgenic mammary tumor models metastasize. Among mouse models that do metastasize, most are derivatives of the PyMT model, which more closely resembles human Luminal subtype tumors than Basal-like tumors [3]. Metastases were not evident in the sternum, liver, or spleen of these mice. A primary cell line established from a TBP tumor formed secondary tumors following serial transplantation into syngeneic (FVB) mammary fat pads (Patel, unpublished), confirming the malignant capacity of TBP tumors.

In summary, TBP tumors displayed heterogeneous histology, including high grade, central necrosis, metaplasia, pushing boarders, and metastasis, features that are characteristic of human *BRCA1*-mutated and Claudin-low breast tumors.

### Global gene expression analysis

**Figure 5:**
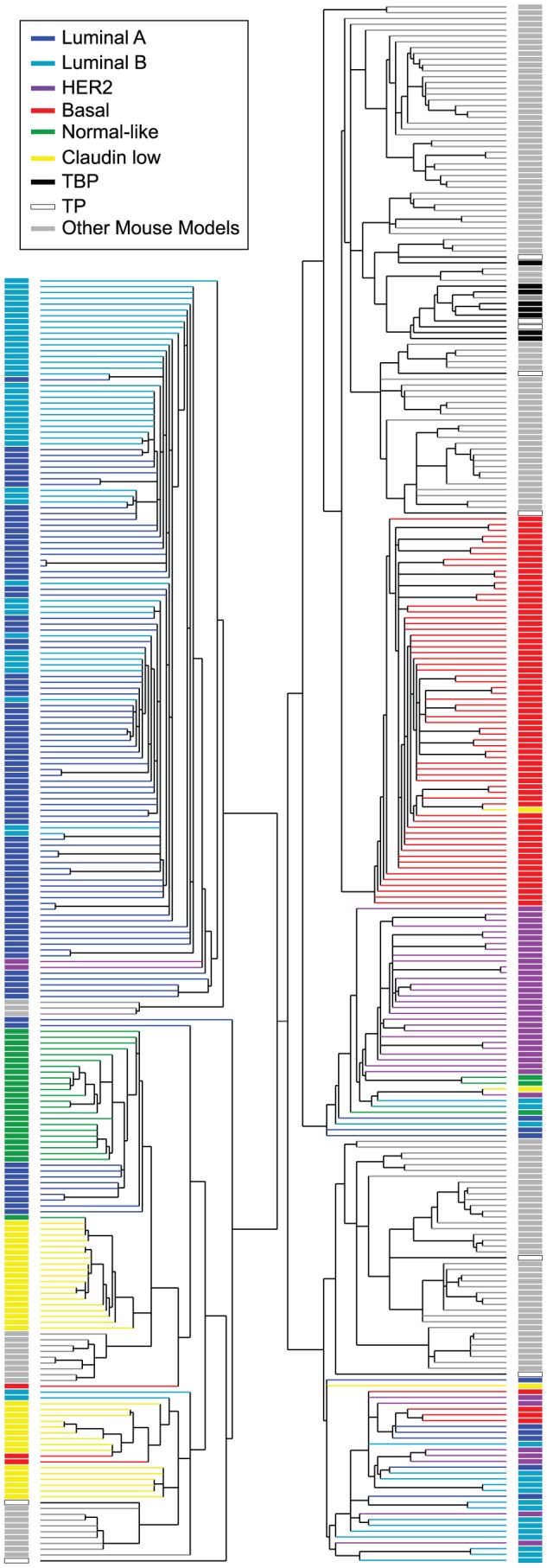
Cross-species comparison of breast cancers. TBP (black boxes, n = 8) and TP (open black boxes, n = 9) tumors were compared to published mouse (gray boxes, n = 135) and human (n = 337) microarray expression profiles (colored according to PAM50 subtype). Most (76%) of our *TgMFT_121_* mouse tumors cluster with human Basal-like breast cancers (red boxes). The Treeview files of the clustering analysis are available in File S2.

The majority of the *TgMFT_121_* mouse tumors (76%) co-segregated with human Basal-like breast cancers by hierarchical clustering of the top ∼1000 most variable genes in a combined data set of mouse (n = 135) and human (n = 337) tumor expression profiles (Figure 5, Figure S1, File S2). Using a similar approach that we reported previously [3], we assayed global transcript levels by microarray using tumors derived from TBP (n = 8) and TP mice (n = 9), and we compared them directly to tumors and normal tissue from other mouse models [3] and patient samples [4]. Two TP tumors clustered with human tumors that showed a mixture of the PAM50 molecular subtypes that were assigned by Prat and colleagues (2010). The two remaining TP tumors clustered with Claudin-low subtype human tumors.

**Figure 6:**
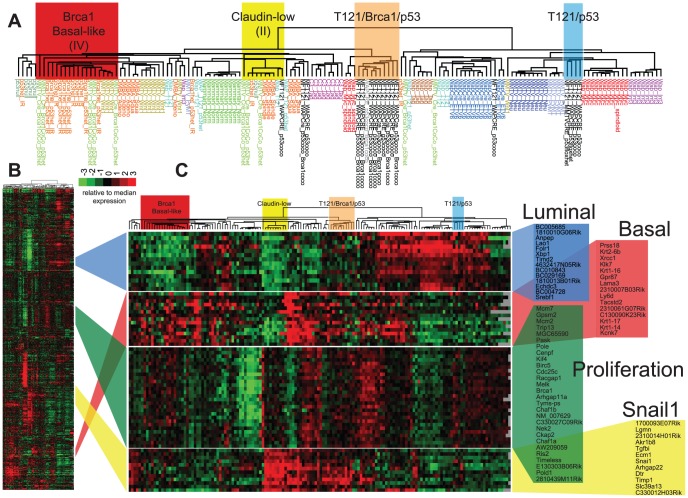
TBP tumors share features of Basal-like and Claudin-low expression signatures. (A) Expression of 866 reference genes of TP (n = 9) and TBP (n = 8) tumors and 13 models of breast cancer. Tumor annotations of the expanded dendrogram (A) are color coded by tumor model. Selected gene clusters (C) correspond to the full data matrix (B). TBP tumors (orange box) cluster with mouse tumors that more closely resemble TNBC on the left branch. 56% (5 of 9) of TP tumors clustered with Luminal-B tumors (blue box). TBP tumors show high expression of *Snai1*-correlated genes in the Claudin-low cluster. The Treeview files of the clustering analysis are included in Table S1 in File S1.

The effect of *Brca1* mutation on TP tumors was more evident when we focused our analysis on the mouse specimens alone (Figure 6, File S3). 56% of the TP tumors (5 of 9) clustered with mouse tumors that we previously showed resemble human Luminal tumors [3]. *Brca1* mutation shifted the tumor phenotype (p = 0.0529, Fisher's exact). All eight TBP tumors segregated with mouse tumors, including other *Brca1*-mutant models that more closely resemble human Basal-like TNBC (Figure 6A). TBP tumors and the other Basal-like mouse tumors expressed low levels of luminal markers and high levels of both Proliferation and Basal cluster transcripts, including *Keratins-14, -6b, -17* (Figure 6C). In contrast, the TP tumors that clustered with Luminal-B-like tumors (Figure 5A, blue box) showed higher expression of luminal marker genes that correlate with the estrogen pathway target *Xbp1* (Figure 5A). Interestingly, TBP tumors were distinct from most other Basal-like mouse tumors in their elevated expression of a subset of Claudin-low signature genes [3], [4], including *Snail1*, *Tgfbi*, *Dtr*, and *Timp1* (Figure 6C).

Four TP tumors (44%) did not segregate with Luminal-like tumors. This finding is consistent with previous reports by us and others that *Rb*/*p53* tumors can also resemble TNBC and the Claudin-low molecular phenotype [3], [18], [20]. A single TP tumor clustered among the previously designated Group II tumors (Figure 6A, yellow box), which are the paradigm cases of the Claudin-low subtype [3]. In addition, a single TP tumor clustered with tumors with a squamous metaplastic histology. Finally, two TP tumors co-segregated with the TBP tumors (Figure 6A, orange box), which is not surprising given that *Rb* is one of the most frequently deleted loci among *Brca1*/*p53*-mutated mouse tumors [22].

### Pathway analysis

**Figure 7:**
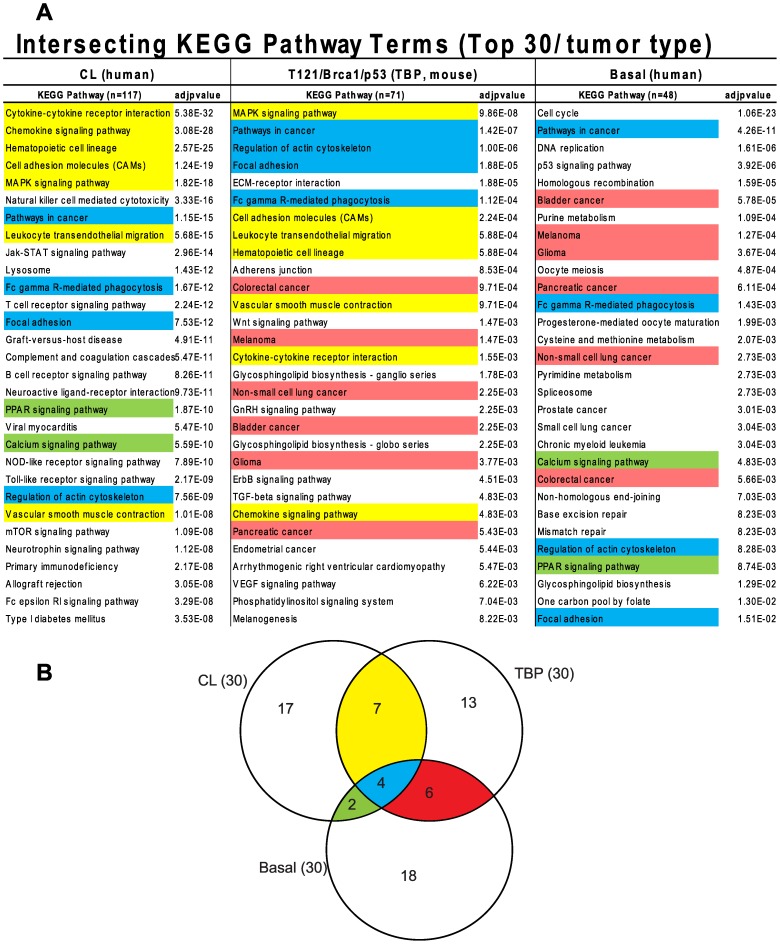
Intersecting KEGG pathway terms. (A) The genes differentially expressed among mouse TBP tumors show enrichment of KEGG pathways associated with human Claudin-low and Basal-like tumors. (B) The pathway terms are colored according to the intersections depicted in the Venn diagram. The full KEGG pathway lists are included in Table S1 in File S1.

The similarity between TBP tumors and human Claudin-low and Basal-like cancers was also evident from pathway analysis of up-regulated genes of each of the three tumor types (Figure 7A, File S1). We queried the KEGG (Kyoto Encyclopedia of Genes and Genomes) and GO (Gene Ontology) databases with lists of genes that were differentially expressed by TBP tumors (see Methods) and by human Claudin-low and Basal-like tumors [4]. Cytokine, chemokine, and MAPK signaling pathways ranked highly among both Claudin-low and TBP tumors. Pathways that are enriched in cancers of diverse origins ranked highly in both Basal-like and murine TBP tumors.

The GO terms associated with the respective tumor types were consistent with the enriched KEGG pathways. Cell-cycle progression (GO:0007049, p = 2.43551E-59) and DNA repair (GO:0034984, p = 6.95081E-22) dominate the list of functions enriched in Basal-like tumors (File S1). Similarly, regulation of cell proliferation (GO:0042127, p = 6.01E-13) is among the top terms for TBP tumors. The three top scoring, inter-related GO terms for TBP tumors are regulation of developmental process (GO:0050793, p = 7.50E-16), organ morphogenesis (GO:0009887, p = 3.53E-14), and tissue development (GO:0009888, p = 1.36E-13). These GO terms are reflective of the enrichment of the *Wnt*, *ErbB*, *TGF-β*, and *VEGF* signaling pathways identified by KEGG pathway analysis. Claudin-low tumors are enriched for wound (GO:0009611, p = 4.29939E-66) and inflammatory responses (GO:0006954, p = 1.26817E-50), which are also among the top functions associated with TBP tumors (7.37E-13 and 6.46E-12, respectively).

### CGH analysis

Given the requirement for BRCA1 in DNA damage repair and centrosome regulation, we tested the hypothesis that TBP tumors harbor more genomic copy number aberrations (CNAs) than do TP tumors with intact *Brca1*. We enumerated CNAs by counting “copy number transitions,” the number of changes in the CGH profile from one copy number level to another that occur within chromosomes [34]. Unexpectedly, we found no statistically significant difference (p = 0.8374) in the mean number of CNAs between TBP tumors (n = 8) and TP tumors (n = 10) using array-based comparative genomic hybridization (aCGH).

**Figure 8:**
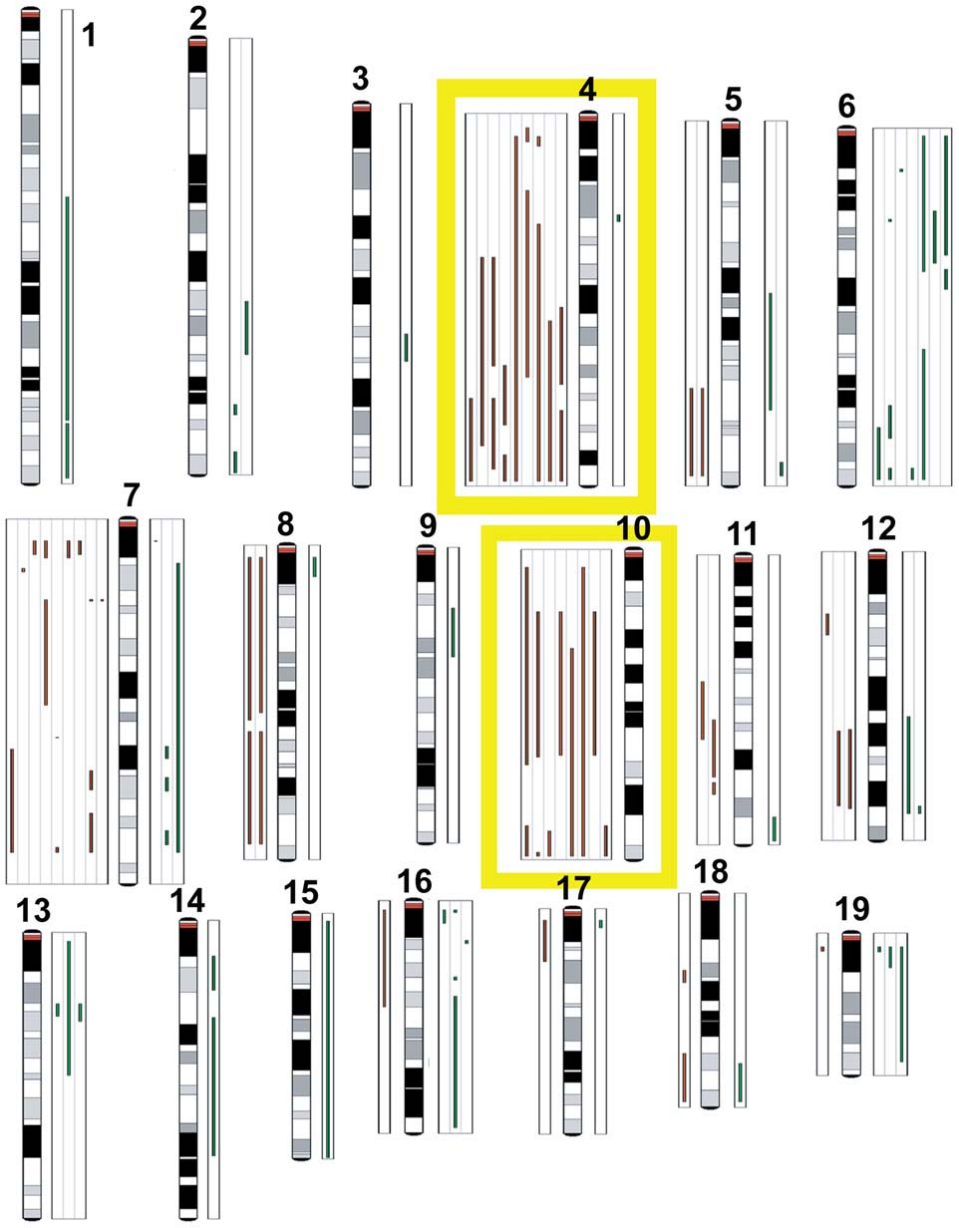
Comparative genomic hybridization. TBP tumors (n = 8) and TP tumors (n = 10) were analyzed by array CGH to identify copy number aberrations (CNAs). Green lines to the right of the chromosome ideograms indicate gains and red lines to the left indicate losses of individual tumor samples. No significant difference was found between the average number of CNAs between TBP and TP tumors (p = 0.8374). Frequent losses were seen on chromosomes 4 and 10. Other recurrent losses included chromosome 7. Frequent gains were associated with chromosome 6.

The low multiplicity of TBP and TP tumors (1–3 per mouse) and their latency indicate that combinations of pRb_f_, Brca1, and p53 pathway perturbations are not sufficient for malignant transformation in our models. To identify potentially collaborating oncogenic events, we manually curated loci with copy number changes (see Methods). In nine tumors (50%), we observed recurrent losses of large, variable regions spanning *chr4* and *chr10* (Figure 8). Both chromosomes harbor many potential tumor suppressors, including regulators of cell death, such as *Tm2d1*, *Utp11l*, *Trp73*, *Dffa*, *Runx3*, *Lck*, *Dhcr24*, *Faf1*, *Pax7*, and *Casp9*, and effectors of cell death, such as *Col18a1*, *Gadd45b*, *Dapk3*, and *Casp14*. Among all the tumors assayed (n = 18), we identified nearly five-hundred loci (Table S9 in File S1) with potential copy number gains. Approximately half of the genes are included on curated lists of cancer-associated genes, including the Cancer Gene Census (Sanger Institute) and the KEGG Pathways in Cancer. We observed focal amplification of several canonical proto-oncogenes, including *c-Myc* amplification (log_2_ratio = 3.64, p<0.0001) in a single TP tumor, *H-ras* amplification in two of ten TP tumors, and *K-ras* amplification in two of eight TBP tumors. Pathway analysis of these five-hundred putative collaborating genes revealed enrichment of several signaling pathways, including the MAP Kinase, Focal Adhesion, Wnt, and ErbB pathways (Table S10 in File S1).

## Discussion

Here we report a highly penetrant engineered mouse model of TNBC. Our previous work showed that when *pRb_f_* and *p53* are simultaneously perturbed in mammary epithelium, adenocarcinomas develop with long latency, suggesting a requirement for additional oncogenic events. However, these mouse tumors displayed only limited chromosomal copy number aberrations [19]. Because genomic instability is a hallmark of malignant transformation [35], especially among *BRCA1* familial cancers [36] and aggressive sporadic breast cancers [37], we hypothesized that *Brca1* mutation would accelerate the tumor development we observed following dual inactivation of *pRb_f_* and *p53*. Our results show that concomitant inactivation of all three tumor suppressor pathways in mammary epithelium has an additive effect on tumor latency and predisposes highly penetrant, malignant carcinomas. Although *Brca1* inactivation accelerated tumorigenesis compared to TP tumors, we observed no statistical difference between chromosomal copy number transitions in tumors with or without *Brca1*, despite the extensive CNAs observed by others in Brca1/p53 tumors [22].

The pRb-regulated cell cycle network is frequently disrupted in TNBC tumors [7], [26], [37], [38], and the *Rb* locus is among the most frequently lost in Brca1/p53 mouse tumors [22], indicating that there is strong selective pressure for *Rb* pathway inactivation. We speculate that direct inactivation of pRb_f_ by T_121_ may allow TBP cells to escape this rate-limiting barrier of transformation without accruing numerous chromosomal aberrations. Thus, in the context of defective pRb and p53 function, tumor progression may be unrelated to the proportion of the genome altered by copy number alterations. It will be important to determine the effect of *Brca1* loss on the abundance and identities of somatic mutations that are not detectable by CGH.

The importance of p53 mutation in breast cancers is well documented and is confirmed in the present study. The dual inactivation of *pRb_f_* and *Brca1* caused markedly increased cell death that was reduced by p53 mutation. p53-*independent* cell death likely remains a significant barrier to tumor progression among TBP tumors and may account, in part, for the observed loss of genomic regions that harbor cell death regulatory genes, most notably on *chr4* where the *p53* paralog *p73* resides. Identifying the genomic alterations that are conserved across species will be useful in evaluating the impact of the myriad of CNAs observed in breast cancers and may help to explain the heterogeneity of TNBCs.

*Brca1* mutation not only accelerated tumor development but also shifted the tumor spectrum. Whereas *T_121_*/*p53* mouse tumors often resembled the Luminal-B molecular subtype breast cancers, which show relatively abundant expression of luminal epithelial cell differentiation markers, TBP tumors consistently shared features of Basal-like and Claudin-low molecular subtypes. Others have argued that Basal-like and Claudin-low gene expression signatures reflect progenitor and stem cell phenotypes, respectively [4], [16], consistent with a role for *Brca1* in mediating stem/progenitor cell maturation [15]. Loss of BRCA1 activity may also alter tumor phenotype through deregulation of the EMT inducer SLUG [39].

The CGH analysis of our mouse tumors revealed CNAs consistent with mutations observed in genomic surveys of human breast cancers [40], [41]. Similar to the studies of human tumors, we saw increased copy numbers of known oncogenic driver genes, including *myc*, *egfr*, *crebbp*, *jak1*, *H-ras*, and *K-ras*, as well as enrichment of pathways implicated in tumor progression, including the WNT signaling pathway, regulation of actin cytoskeleton, focal adhesion, cell shape, and mobility proteins. Far fewer investigations have focused on genetic deletions and cancer development mechanisms. We also found decreased copy numbers of known tumor suppressors, including *map2k*, *ppp2r*, and *pten*. Given the strong similarities between our mouse model and aggressive human breast cancers, the TBP model provides an invaluable preclinical platform to identify and assess potential therapeutics for aggressive and chemoresistant breast cancer subtypes [42], [43].

## Materials and Methods

### Ethics statement

This study was performed in strict accordance with the recommendations in the Guide for the Care and Use of Laboratory Animals of the National Institutes of Health.

### Derivation of *MFT_121_* transgenic mice

The LoxP-eGFP-Stop-LoxP cassette and T_121_-encoding DNA were cloned into EcoRI HindIII sites of MMTV-SV40-Bssk (Addgene plasmid 1824). The LoxP-eGFP-Stop-LoxP cassette was provided courtesy of the T. Jacks lab. Resulting and subsequent generation *MFT* ***_121_*** transgenic mice were identified by PCR amplification of a 215-bp fragment using the oligo pair: 5′-GCATCCAGAAGCCTCCAAAG -3′ and 5′-GAATCTTTGCAGCTAATGGACC-3′ complementary to the T_121_ sequence. Cre transgenic mice were identified using the oligo pair: 5′-TGATGAGGTTCGCAAGAACC-3′ and 5′-CCATGAGTGAACGAACCTGG-3′. The cycling profile was 94°C for 2 min., 35 cycles of 94°C for 20 sec., 62°C for 45 sec., and 72°C for 45 sec.; the final incubation of 72°C was for 2 min. We established five *TgMFT_121_* founder transgenic lines, though three lines failed to express the eGFP reporter. We describe here our studies of the single mouse line with higher eGFP expression in virgin mammary glands. eGFP expression was also evident in salivary glands and foot pads in this line (data not shown).

### Transgenic breeding strategies

*TgMFT_121;_TgWAP-Cre* mice were mated to *p53* conditional allele mice (*p53^f/f^*) [44]. *p53* genotypes were determined by PCR using two reactions: neomycin primer 5′-TCCTCGTGCTTTACGGTATC-3′, *p53* primer 5′-TATACTCAGAGCCGGCCT-3′, 525-bp product; the endogenous *p53* allele; substituting 5′-ACAGCGTGGTGGTACCTTAT-3′ for the *neo* primer, 475-bp product. Cycling parameters were the same as they were for the T_121_ reaction. We produced female mice with the genotypes *TgMFT_121;_TgWAP-Cre;p53* ^f/+^ and *TgMFT_121;_TgWAP-Cre;p53^f/f^*, and female littermates served as controls. To study the effect of *Brca1* loss, *TgMFT_121_*; *TgWAP-Cre* mice were mated to *Brca1* ***^f/f^***,*p53^f/f^* mice [44]. *Brca1* genotypes were determined by PCR using two reactions. We generated female mice with the genotypes *TgMFT_121_*; *TgWAP-Cre; Brca1* ***^f/+^*** *,p53* ^f/+^ and *TgMFT_121_*; *TgWAP-Cre; Brca1* ***^f/f^*** *,p53* ^f/f^ with nontransgenic (Cre negative) littermate controls for each cohort. Pregnancy induced *WAP-Cre* transgene expression. Parturition of the first litter was designated as Day 1 for all aging studies. Matings with *TgMMTV-Cre* mice (Line F) yielded small litter sizes; therefore, experiments reported here employed *TgWap-Cre* unless otherwise indicated.

### Histopathology and apoptosis assays

A portion of each mammary sample was fixed overnight in 10% phosphate-buffered formalin, transferred to 70% ethanol, and then embedded in paraffin. Samples were sectioned for 10 successive layers at 5-µm intervals and stained with hematoxylin and eosin for histopathologic examination, as described previously. Apoptosis levels were assessed using the terminal deoxynucleotidyl transferase–mediated dUTP-biotin nick end labeling (TUNEL) method with standard protocols. Differences in apoptosis levels between mice with different genotypes were evaluated by the *t* test (*p*<0.05 was deemed statistically significant).

### Immunostaining

Immunohistochemical analysis was performed using formalin-fixed paraffin sections. Antigen retrieval for all antibodies was done by boiling the slides in citrate buffer (pH 6.0) for 15 min. Antibodies were α-cytokeratins 8/18 (Ker8/18, 1∶450 Progen, GP11), α-cytokeratin 5 (K5, 1∶8000, Covance, PRB-160P), smooth muscle actin (SMA; 1∶1,000, mouse A2537; Sigma, St. Louis, MO), anti–phosphorylated histone H3 (1∶100, rabbit 06-570; Upstate, Waltham, MA) and SV40 (monoclonal Ab2, 1∶100; Oncogene, Cambridge, MA). All immunofluorescence reactions were done using AlexaFlour-conjugated secondary antibodies (AlexaFluor 488 and 594, Molecular Probes). Slides were counterstained with 4′,6-diamidino-2-phenylindole (DAPI) using Hardset Mounting Media (Vector Laboratories).

### Microarray analysis

We compared microarray profiles of T_121_/p53 (n = 9) and TBP (n = 8) tumors to published microarray profiles (n = 152) using two-way hierarchical clustering (centroid linkage) of 866 “intrinsic genes” [3], [45]. Total RNA was collected from end-stage tumors. RNA was purified using the Qiagen RNeasy Mini Kit according to the manufacturer's protocol using 20–30 mg tissue. RNA integrity was assessed using the RNA 6000 Nano LabChip by Bioanalyzer (Agilent). Two micrograms of total RNA were reverse transcribed, amplified, and labeled with Cy5 using a Low RNA Input Amplification kit (Agilent). Common reference RNA consisted of total RNA harvested from equal numbers of C57Bl6/J and 129 male and female Day 1 pups (courtesy of Dr. Cam Patterson, UNC). Reference RNA was reverse transcribed, amplified, and labeled with Cy3. The amplified sample and reference were co-hybridized overnight to Agilent Mouse Oligo Microarrays (G4121A). They were then washed and scanned on an Axon GenePix 4000B scanner, analyzed using GenePix 4.1 software, and uploaded into the UMD database (https://genome.unc.edu/) where Lowess normalization is automatically performed. All data were submitted to GEO (GSE34479). The genes for all analyses were filtered by: 1) requiring intensity values in both channels to have a mean Lowess normalized intensity of >10, 2) Values being reported in >70% of the samples, and 3) the absolute value of the log_2_ of the ratio of Channel 2/Channel 1 for at least three arrays having to be >1.6. Hierarchical clustering was performed using Cluster v3.0 and displayed using JavaTreeview v1.0.8.

We identified 871 differentially expressed TBP transcripts using SAM implemented in BRB-ArrayTools (R. Simon and the BRB-ArrayTools Development Team, NCI; Table 1; FDR 0.0485, delta 0.92931). Gene ontology analyses were performed using the FatiGO tool (Babelomics ver. 4.2, babelomics.bioinfo.cipf.es). Mouse gene symbols were converted to human EntrezIDs using Agilent annotations and the Mouse Genome Informatics database of The Jackson Laboratory for comparisons. The Fisher's exact two-tailed test was used to determine significance of GO (biological process [levels 3–9]) and KEGG pathway terms. Terms with p<0.05 are reported.

### Combined murine and human expression data sets

For the mouse tumor data set, IDs for 21,670 unfiltered probes were retrieved (GSE34479). Human EntrezIDs were assigned to orthologous genes as above. Mean values of redundant mouse probes were calculated, missing values were imputed, the columns were standardized to N(0,1), and the rows were median centered using R (ver. 2.10.0). For the human data set, we downloaded the file “UNC337arraydata_imputedCollapsed.txt” [4] from the UNC MicroArray Database (genome.unc.edu). The two data sets were corrected for systemic biases using Distance Weighted Discrimination [46]. The combined data set was used for centroid linkage hierarchical clustering analysis.

### CGH analysis

We performed array CGH essentially as previously described [47]. Briefly, genomic DNA was isolated from tumors, fluorescently labeled, and competitively hybridized with wt DNA spotted BAC (Bacterial Artificial Chromosomes) arrays. All data were submitted to GEO (GSE40925). We used the HaarSeg algorithm with default parameters implemented in waviCGH (wavi.bioinfo.cnio.es) for chromosomal segmentation of mutations and for CNA calling. We manually curated BAC clones spanning putative CNAs with a conservative tumor:normal DNA threshold of log_2_ratio >0.5 or <−0.5. Genes mapping to the BAC clones were identified using the National Center for Biotechnology Map Viewer and the Jackson Laboratory Mouse Genome Database. Gene lists were compared to the Cancer Gene Census (Sanger Institute), the KEGG Pathways in Cancer, Atlas Genetics Oncology, and the Michigan Molecular Interactions database. For the pathway analysis, we used the SAM algorithm implemented in BRB-Array Tools to identify differentially expressed genes (**TBP_SAM**) among TBP tumors versus the rest of the mouse tumors in the data set. We interrogated KEGG (**TBP_KEGG**) and GO (**TBP_GO_BP**) databases using the FatiGO algorithm implemented in the Babelomics (ver. 4.2) suite of bioinformatics tools (babelomics.bioinfo.cipf.es). We compared these results to the differentially expressed genes reported by Prat et al. (2010) of human Claudin-low (**CL_KEGG**, **CL_GO_BP**) and Basal-like (**Basa_KEGG**, **Basal-_GO_BP**) tumors. We also used the FatiGO tool in the Babelomics 4.3.0 integrative platform with parameters set for the Fisher's exact two-tailed test to determine the most alternately expressed KEGG pathways among the amplified genes determined by CGH analysis.

## Supporting Information

Figure S1 Hierarchical clustering of the cross-species analysis summarized in Figure 5.
(PDF)

File S1 Tumor annotations (Table S1 Tumor_Descriptors). Differentially expressed genes defined by SAM analysis of TBP tumors (S2 TBP_SAM). Gene Ontology (GO) terms enriched among tumor TBP, Basal, and Claudin-low subtypes (S3 TBP_GO_BP, S4 Basal_GO_BP, and S5 CL_GO_BP). KEGG pathways enriched among the tumor subtypes (S6 TBP_KEGG, S7 Basal_KEGG, S8 CL_KEGG). Among all tumors assayed (n = 18), we identified nearly five-hundred loci with potential copy-number gains (log_2_ratio >0.5, Table S9 putative_gained_CGH_loci). Approximately half of the genes are previously defined oncogenes represented in oncogene lists including the Cancer Gene Census (Sanger Institute) and the KEGG Pathways in Cancer. The KEGG Pathways enriched among putative gained genes (Table S10 CGH_Gain_KEGG).
(XLSX)

File S2 JavaTreeview data files of the cross-species clustering analysis shown in Figure 5.
(ZIP)

File S3 JavaTreeview data files of the mouse tumor clustering analysis shown in Figure 6.
(ZIP)
