# Supplementary material for: Cooperativity of Rb, Brca1, and p53 in Malignant Breast Cancer Evolution
Source: PLoS Genet. 2012 Nov 15;8(11):e1003027. doi: 10.1371/journal.pgen.1003027 (PMC3500050; doi:10.1371/journal.pgen.1003027)

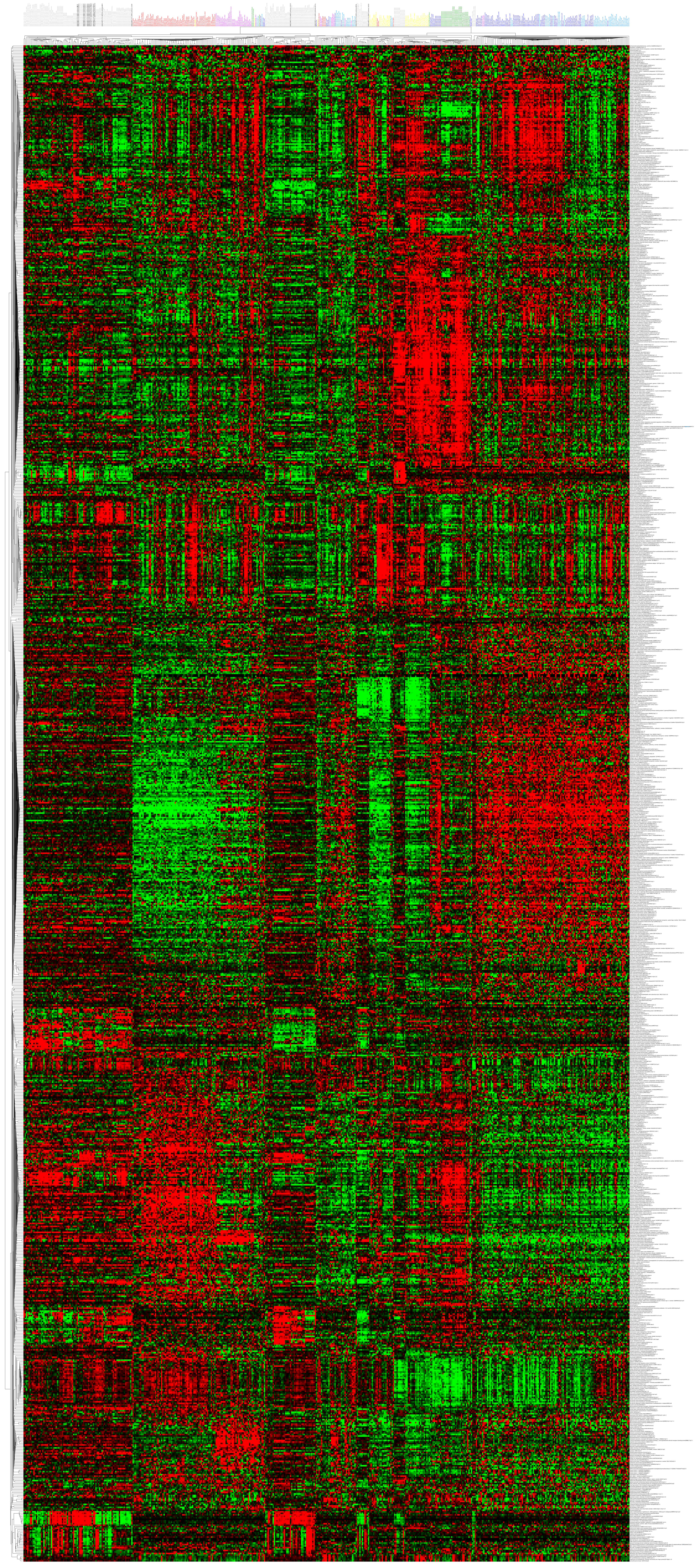

Supplement: Figure S1 — Hierarchical clustering of the cross-species analysis summarized in Figure 5. (PDF) [file pgen.1003027.s001.pdf]
